# Supplementary material for: Genomic surveillance reveals a dengue 2 virus epidemic lineage with a marked decrease in sensitivity to Mosnodenvir
Source: Nat Commun. 2024 Oct 9;15:8667. doi: 10.1038/s41467-024-52819-z (PMC11464713; doi:10.1038/s41467-024-52819-z)

Supplementary Information

Supplementary Table 1: Prevalence among sequences from the French Caribbean Islands for described JNJ A07 resistance mutations. The percentage of sequences exhibiting the mutation is provided for each mutation.

NS4B mutation	Prevalence in French Caribbean Islands sequences (%)
**F47Y**	0
**S85L**	0
**V91A**	100
**L94F**	0
**P104S**	0
**T108I**	0
**A137T**	0
**T216N**	0
**T216P**	0

Supplementary Table 2: TMRCA estimates from BEAST analyses under different substitution models and coalescent tree priors. Shown for each coalescent tree prior is the median, with the 95% highest probability distribution of TMRCA in parentheses. Also shown is the log marginal likelihood obtained using path-sampling and stepping-stone sampling for each model/prior combination.

Substitution model	Tree prior	Median	95%HPD	Log marginal likelihood (PS)	Log marginal likelihood (SS)
HKY+G4	Constant	2021-08-27	[2020-08-27;2022-05-25]	-16310.3487763015	-16310.1637889435
SRD06	Constant	2021-09-05	[2020-09-01;2022-05-31]	-16240.6171825888	-16240.8058067342
HKY+G4	Exponential growth	2022-03-14	[2021-08-17;2022-09-03]	-16294.5233796415	-16294.4161644864
SRD06	Exponential growth	2022-03-16	[2021-08-30;2022-09-25]	-16228.0096766946	-16228.1267008773
HKY+G4	Skygrid	2022-04-01	[2021-08-01;2022-12-01]	-16291.3119427217	-16291.4397508622
SRD06	Skygrid	2022-04-01	[2021-08-01;2022-11-26]	-16221.3518324382	-16221.2804724036

Supplementary Table 3: DENV-3 V91A-carrying sequences information. For each DENV-3 sequence carrying mutation V91A, the Genbank accession number, geographic origin, collection year, and genotype are provided.

Accession number	Geographic origin	Year	Genotype
EU660410	Viet Nam	2006	II
MZ008477	Nicaragua	2013	III
MZ008475	Nicaragua	2013	III
MW946935	Thailand	1997	II
MW946852	Thailand	2006	II
MW946829	Thailand	2005	II
KU509284	Thailand	2008	II
KF973486	Nicaragua	2012	III
KF921927	Nicaragua	2010	III
OQ821525	Cuba	2022	III

Supplementary Table 4. Ct values obtained for clinical isolates

Sample Number	Ct
65877	21
72674	28
73058	25
74234	30

Supplementary Table 5. Key resources table

REAGENT or RESOURCE	SOURCE	IDENTIFIER
Chemicals, peptides, and recombinant proteins		
Minimal essential medium	ThermoFisher Scientific	Cat#21090022
Non-essential amino acids	ThermoFisher Scientific	Cat#11140035
Penicillin/Streptomycin	ThermoFisher Scientific	Cat#15140122
Heat-inactivated fetal bovine serum	ThermoFisher Scientific	Cat#10270098
Critical commercial assays		
QIAamp 96 DNA kit	Qiagen	Cat#51331
GoTaq 1 step RT-qPCR kit	Promega	Cat#A6020
Software and algorithms		
GraphPad Prism 9 software	GraphPad software	https://www.graphpad.com/
QuantStudio 12K Flex Real-Time PCR Software v.1.2.3	Applied Biosystems	https://www.thermofisher.com/fr/fr/home/global/forms/quantstudio-12k-flex-software-download.html

Supplementary Data 1: Sequence information for the 98 sequences from the French Caribbean Island epidemic included in the analysis. For each sequence, the Genbank accession number, strain name and collection date are provided.

Supplementary Data 2: DENV-2 V91A-carrying sequences information. For each DENV-2 sequence carrying mutation V91A, the Genbank accession number, geographic origin, collection year, and genotype are provided.

Supplementary Data 3. Data availability and snapshot statements for GISAID DENV2 sequences.

Supplementary Data 4 : Source data for Figure 1. This table is provided as a supplementary file. Detailed EC_{50} and EC_{90} values for JNJ-A07 and JNJ-1802.

Supplementary Figure 1: Subtree including the French Caribbean Islands epidemic lineage. The subtree has been extracted from the maximum-likelihood phylogeny including all Genbank and GISAID sequences from DENV-2 genotype II covering more than 85 % of the CDS available on Genbank as of March 2024 and a set of reference sequences from other genotypes. Phylogenetic inference was performed using IQTREE2 under a GTR+F+R5 substitution model with ultrafast bootstrap approximation (1000 replicates). Sequences from the French Caribbean Islands epidemic are highlighted in sea green, sequences from the USA (Florida) are colored in mustard yellow, the sequence from Brazil is colored in olive green and sequences from other locations are colored in grey.

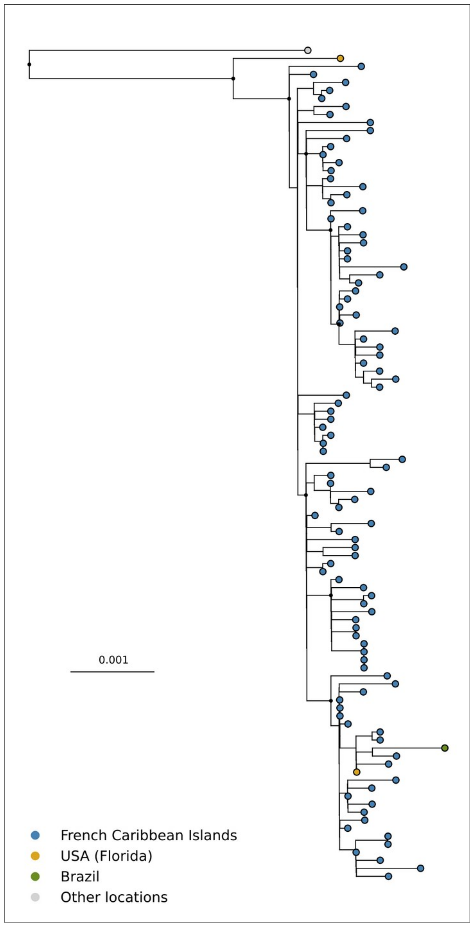

Supplementary Figure 2: DENV-2 V91A-carrying sequences genotypes. Maximum-Likelihood phylogeny of all V91A-carrying DENV2 sequences with a set of reference sequences representative of DENV-2 genotypes Phylogenetic inference was performed using IQTREE2 under the best substitution model identified by ModelFinder with ultrafast bootstrap approximation (1000 replicates). The tree was rooted using the highly divergent strain QML22 (KX274130). Nodes with boostrap support above 95 are highlighted with a black circle.

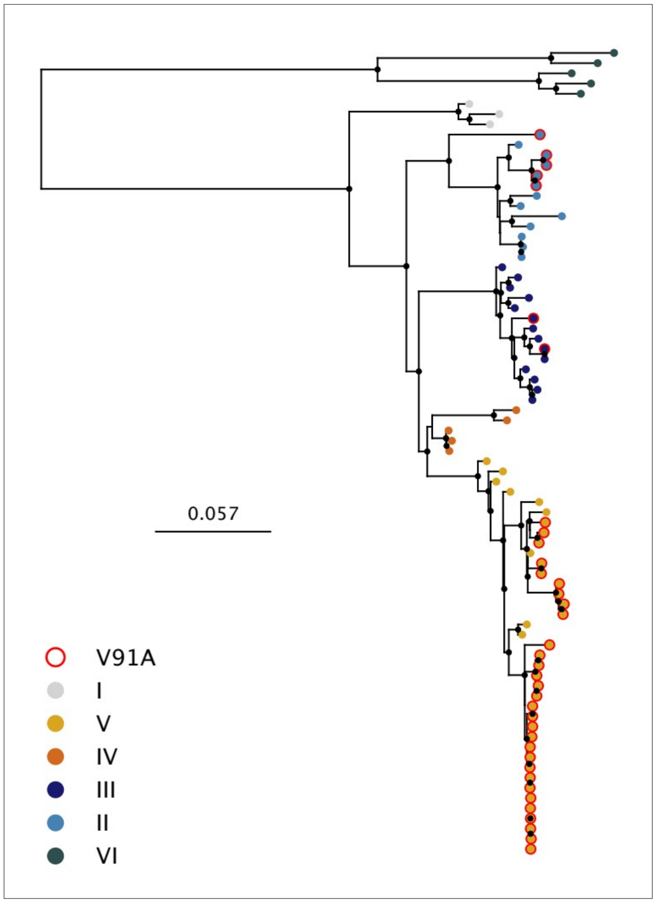

Supplementary Figure 3: DENV-3 V91A-carrying sequences genotypes. Maximum-Likelihood phylogeny of all V91A-carrying DENV2 sequences with a set of reference sequences representative of DENV-3 genotypes Phylogenetic inference was performed using IQTREE2 under the best substitution model identified by ModelFinder with ultrafast bootstrap approximation (1000 replicates). The tree was midpoint rooted. Nodes with boostrap support above 95 are highlighted with a black circle.

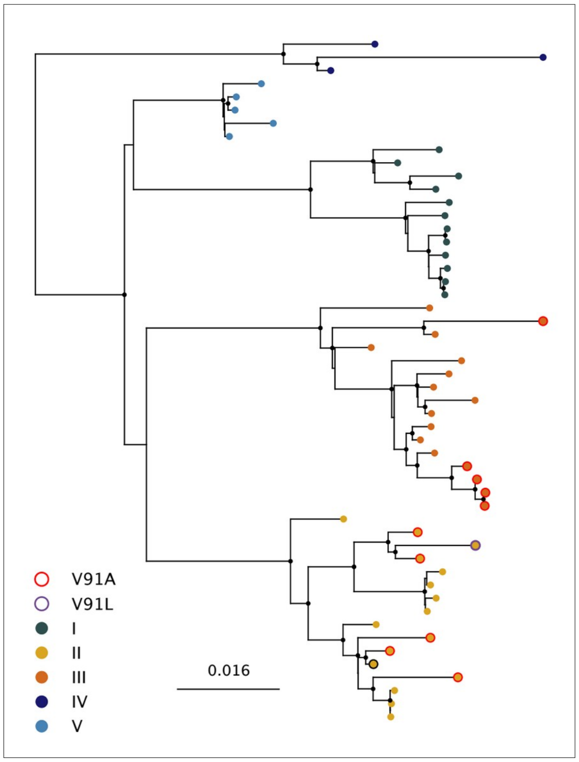

Supplementary Figure 4: Root-to-tip analysis of sequences used for bayesian inference. Regression of genetic distance against time for all sequences included in the dataset used for bayesian inference.

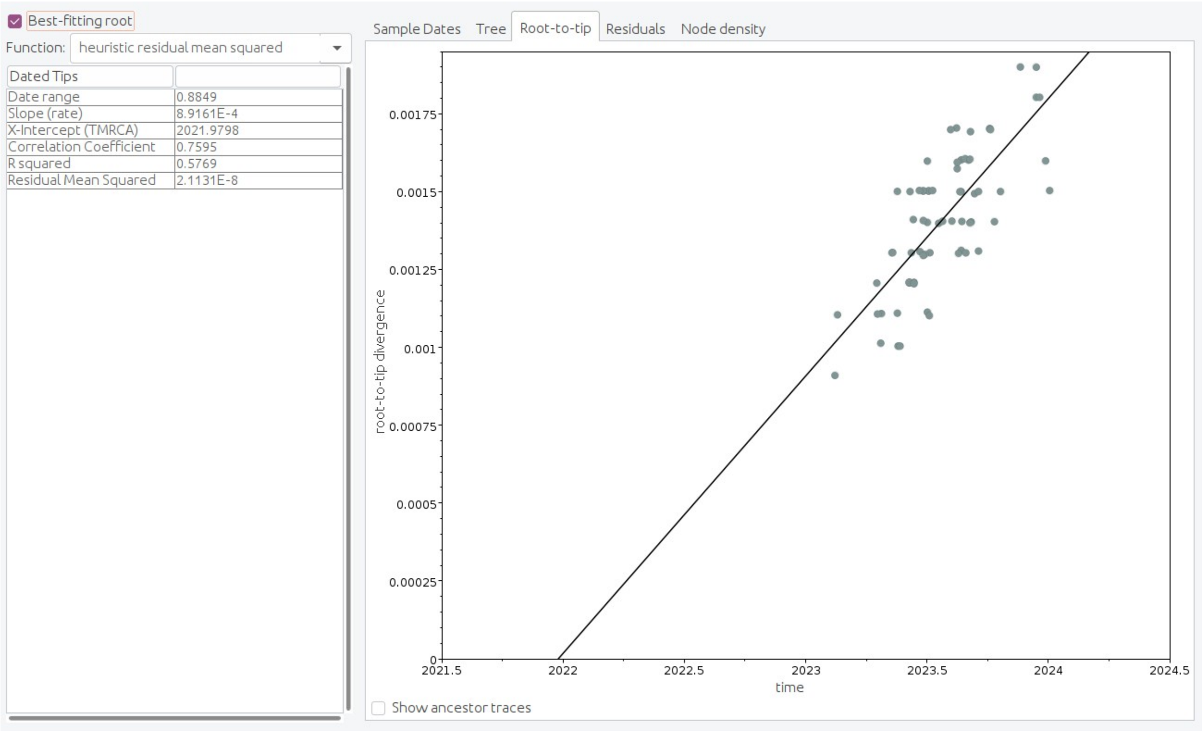

Supplement: Supplementary file 1 — Supplementary Information [file 41467_2024_52819_MOESM1_ESM.pdf]
