# Supplementary material for: Genomic surveillance reveals a dengue 2 virus epidemic lineage with a marked decrease in sensitivity to Mosnodenvir
Source: Nat Commun. 2024 Oct 9;15:8667. doi: 10.1038/s41467-024-52819-z (PMC11464713; doi:10.1038/s41467-024-52819-z)
Supplement: Supplementary file 3 — Description of Additional Supplementary Files [file 41467_2024_52819_MOESM3_ESM.pdf]

Description of Additional Supplementary Files

File Name: Supplementary Data 1
Description: Sequence information for the 98 sequences from the French Caribbean Island epidemic included in the analysis. For each sequence, the Genbank accession number, strain name and collection date are provided.

File Name: Supplementary Data 2
Description: DENV-2 V91A-carrying sequences information. For each DENV-2 sequence carrying mutation V91A, the Genbank accession number, geographic origin, collection year, and genotype are provided.

File Name: Supplementary Data 3
Description: Data availability and snapshot statements for GISAID DENV2 sequences.

File Name: Supplementary Data 4
Description: Source data for Figure 1. This table is provided as a supplementary file. Detailed EC50 and EC90 values for JNJ-A07 and JNJ-1802.
