# Supplementary material for: Genomic surveillance reveals a dengue 2 virus epidemic lineage with a marked decrease in sensitivity to Mosnodenvir
Source: Nat Commun. 2024 Oct 9;15:8667. doi: 10.1038/s41467-024-52819-z (PMC11464713; doi:10.1038/s41467-024-52819-z)
Supplement: Supplementary file 6 — Supplementary Data 3 [file 41467_2024_52819_MOESM6_ESM.pdf]

SUPPLEMENTAL TABLE

Data Availability

GISAID Identifier:	EPI_SET_240902um
doi:	10.55876/gis8.240902um

All genome sequences and associated metadata in this dataset are published in GISAID's EpiArbo database. To view the contributors of each individual sequence with details such as accession number, Virus name, Collection date, Originating Lab and Submitting Lab and the list of Authors, visit 10.55876/gis8.240902um

Data Snapshot

- EPI_SET_240902um is composed of 781 individual genome sequences.
- The collection dates range from 2023-01-05 to 2024-04-17;
- Data were collected in 15 countries and territories;
- All sequences in this dataset are compared relative to the official reference sequence employed by GISAID.
