# Supplementary material for: Genomic surveillance reveals a dengue 2 virus epidemic lineage with a marked decrease in sensitivity to Mosnodenvir
Source: Nat Commun. 2024 Oct 9;15:8667. doi: 10.1038/s41467-024-52819-z (PMC11464713; doi:10.1038/s41467-024-52819-z)
Supplement: Supplementary file 8 — Reporting Summary [file 41467_2024_52819_MOESM8_ESM.pdf]

nature portfolio

Corresponding author(s): Raphaelle Klitting and Franck Touret

Last updated by author(s): Sep 6, 2024

Reporting Summary

Nature Portfolio wishes to improve the reproducibility of the work that we publish. This form provides structure for consistency and transparency in reporting. For further information on Nature Portfolio policies, see our Editorial Policies and the Editorial Policy Checklist.

Statistics

For all statistical analyses, confirm that the following items are present in the figure legend, table legend, main text, or Methods section.

n/a	Confirmed	
☐	☒	The exact sample size (*n*) for each experimental group/condition, given as a discrete number and unit of measurement
☐	☒	A statement on whether measurements were taken from distinct samples or whether the same sample was measured repeatedly
☒	☐	The statistical test(s) used AND whether they are one- or two-sided<br>*Only common tests should be described solely by name; describe more complex techniques in the Methods section.*
☒	☐	A description of all covariates tested
☒	☐	A description of any assumptions or corrections, such as tests of normality and adjustment for multiple comparisons
☐	☒	A full description of the statistical parameters including central tendency (e.g. means) or other basic estimates (e.g. regression coefficient) AND variation (e.g. standard deviation) or associated estimates of uncertainty (e.g. confidence intervals)
☒	☐	For null hypothesis testing, the test statistic (e.g. *F*, *t*, *r*) with confidence intervals, effect sizes, degrees of freedom and *P* value noted<br>*Give P values as exact values whenever suitable.*
☐	☒	For Bayesian analysis, information on the choice of priors and Markov chain Monte Carlo settings
☒	☐	For hierarchical and complex designs, identification of the appropriate level for tests and full reporting of outcomes
☒	☐	Estimates of effect sizes (e.g. Cohen's *d*, Pearson's *r*), indicating how they were calculated

Our web collection on statistics for biologists contains articles on many of the points above.

Software and code

Policy information about availability of computer code

Data collection	Automated RNA extractions were performed on the QIAcube HT automat (Qiagen). For RT-qPCR, a QuantStudio 12K Flex Real-Time PCR System (Applied Biosystems) was used.
Data analysis	The alignment of genomic sequences was made using the program MAFFT version 7 (https://mafft.cbrc.jp/alignment/server/) and manually edited using the program AliView version 1.28 (https://ormbunkar.se/aliview/). Potential recombinant sequences were detected potential recombinant sequences from the DENV-2, DENV-2 Comopolitan and DENV-3 datasets using the Recombination Detection Program (RDP) version 4 (http://web.cbio.uct.ac.za/~darren/rdp.html). Maximum-likelihood phylogenetic inference was performed using IQ-Tree version 1.6.12 (http://www.iqtree.org/). The time-scaled phylogeny was inferred using a Bayesian method implemented in the open-source program BEAST v1.10.4 (https://beast.community/programs) and the BEAGLE library version 4 (https://beast.community/beagle). We used the program Tracer version 1.7 (https://beast.community/tracer) to inspect the convergence and mixing properties of the BEAST outputs. The BEAST xml files of the bayesian analysis, as well as the alignment and tree files for all phylogenetic analyses are available at https://github.com/Snseli/Dengue_resistance_supplementary.<br>RT-qPCR data were analysed using the QuantStudio 12K Flex software (v1.2.3). Inhibition values for antiviral molecules were plotted using GraphPad Prism 9 software (Graphpad software).

For manuscripts utilizing custom algorithms or software that are central to the research but not yet described in published literature, software must be made available to editors and reviewers. We strongly encourage code deposition in a community repository (e.g. GitHub). See the Nature Portfolio guidelines for submitting code & software for further information.

Data

Policy information about availability of data

All manuscripts must include a data availability statement. This statement should provide the following information, where applicable:

- Accession codes, unique identifiers, or web links for publicly available datasets
- A description of any restrictions on data availability
- For clinical datasets or third party data, please ensure that the statement adheres to our policy

All alignment, xml and tree files for this study are available at: https://github.com/Snseli/Dengue_resistance_supplementary.git, https://doi.org/10.5281/zenodo.13680149). All unique sequence IDs are detailed in the supplementary information and on the github repository listed above.
Raw data from the experiments are provided as a supplementary information.

Research involving human participants, their data, or biological material

Policy information about studies with human participants or human data. See also policy information about sex, gender (identity/presentation), and sexual orientation and race, ethnicity and racism.

Reporting on sex and gender	*Use the terms sex (biological attribute) and gender (shaped by social and cultural circumstances) carefully in order to avoid confusing both terms. Indicate if findings apply to only one sex or gender; describe whether sex and gender were considered in study design; whether sex and/or gender was determined based on self-reporting or assigned and methods used. Provide in the source data disaggregated sex and gender data, where this information has been collected, and if consent has been obtained for sharing of individual-level data; provide overall numbers in this Reporting Summary. Please state if this information has not been collected. Report sex- and gender-based analyses where performed, justify reasons for lack of sex- and gender-based analysis.*
Reporting on race, ethnicity, or other socially relevant groupings	*Please specify the socially constructed or socially relevant categorization variable(s) used in your manuscript and explain why they were used. Please note that such variables should not be used as proxies for other socially constructed/relevant variables (for example, race or ethnicity should not be used as a proxy for socioeconomic status). Provide clear definitions of the relevant terms used, how they were provided (by the participants/respondents, the researchers, or third parties), and the method(s) used to classify people into the different categories (e.g. self-report, census or administrative data, social media data, etc.) Please provide details about how you controlled for confounding variables in your analyses.*
Population characteristics	*Describe the covariate-relevant population characteristics of the human research participants (e.g. age, genotypic information, past and current diagnosis and treatment categories). If you filled out the behavioural & social sciences study design questions and have nothing to add here, write "See above."*
Recruitment	*Describe how participants were recruited. Outline any potential self-selection bias or other biases that may be present and how these are likely to impact results.*
Ethics oversight	*Identify the organization(s) that approved the study protocol.*

Note that full information on the approval of the study protocol must also be provided in the manuscript.

Field-specific reporting

Please select the one below that is the best fit for your research. If you are not sure, read the appropriate sections before making your selection.

☐ Life sciences ☐ Behavioural & social sciences ☐ Ecological, evolutionary & environmental sciences

For a reference copy of the document with all sections, see nature.com/documents/nr-reporting-summary-flat.pdf

Life sciences study design

All studies must disclose on these points even when the disclosure is negative.

Sample size	*Describe how sample size was determined, detailing any statistical methods used to predetermine sample size OR if no sample-size calculation was performed, describe how sample sizes were chosen and provide a rationale for why these sample sizes are sufficient.*
Data exclusions	*Describe any data exclusions. If no data were excluded from the analyses, state so OR if data were excluded, describe the exclusions and the rationale behind them, indicating whether exclusion criteria were pre-established.*
Replication	*Describe the measures taken to verify the reproducibility of the experimental findings. If all attempts at replication were successful, confirm this OR if there are any findings that were not replicated or cannot be reproduced, note this and describe why.*
Randomization	*Describe how samples/organisms/participants were allocated into experimental groups. If allocation was not random, describe how covariates were controlled OR if this is not relevant to your study, explain why.*

Blinding | *Describe whether the investigators were blinded to group allocation during data collection and/or analysis. If blinding was not possible, describe why OR explain why blinding was not relevant to your study.*

Behavioural & social sciences study design

All studies must disclose on these points even when the disclosure is negative.

Study description | *Briefly describe the study type including whether data are quantitative, qualitative, or mixed-methods (e.g. qualitative cross-sectional, quantitative experimental, mixed-methods case study).*

Research sample | *State the research sample (e.g. Harvard university undergraduates, villagers in rural India) and provide relevant demographic information (e.g. age, sex) and indicate whether the sample is representative. Provide a rationale for the study sample chosen. For studies involving existing datasets, please describe the dataset and source.*

Sampling strategy | *Describe the sampling procedure (e.g. random, snowball, stratified, convenience). Describe the statistical methods that were used to predetermine sample size OR if no sample-size calculation was performed, describe how sample sizes were chosen and provide a rationale for why these sample sizes are sufficient. For qualitative data, please indicate whether data saturation was considered, and what criteria were used to decide that no further sampling was needed.*

Data collection | *Provide details about the data collection procedure, including the instruments or devices used to record the data (e.g. pen and paper, computer, eye tracker, video or audio equipment) whether anyone was present besides the participant(s) and the researcher, and whether the researcher was blind to experimental condition and/or the study hypothesis during data collection.*

Timing | *Indicate the start and stop dates of data collection. If there is a gap between collection periods, state the dates for each sample cohort.*

Data exclusions | *If no data were excluded from the analyses, state so OR if data were excluded, provide the exact number of exclusions and the rationale behind them, indicating whether exclusion criteria were pre-established.*

Non-participation | *State how many participants dropped out/declined participation and the reason(s) given OR provide response rate OR state that no participants dropped out/declined participation.*

Randomization | *If participants were not allocated into experimental groups, state so OR describe how participants were allocated to groups, and if allocation was not random, describe how covariates were controlled.*

Ecological, evolutionary & environmental sciences study design

All studies must disclose on these points even when the disclosure is negative.

Study description | In our study we performed phylogenetic analyses and in vitro testing to evaluate the circulation of Mosnodenvir-resistant strains of DENV with a focus on the ongoing epidemic in the French Caribbean islands. First, (i) we screened sequences from the current epidemic for previously described JNJ A07 resistance mutations, (ii) evaluated the in vitro efficacy of JNJ A07 and JNJ 1802 (mosnodenvir) against four clinical isolates, and (iii) used Maximum-Likelihood (ML) and bayesian inference to estimate the date of emergence of the V91A-lineage associated with the epidemic. Then, (iv) we screened all publicly available DENV2 sequences for the V91A mutation, performed ML phylogenetic inference to assess past emergence events, and evaluated the pan-genotype effect of the mutation on in vitro resistance to Mosnodenvir in a DENV2 strain from the Asian II genotype. Similarly, (v) we screened all publicly available DENV3 sequences for the V91A mutation, assessed pas emergence events using ML inference, and evaluated the effect of the mutation on in vitro resistance to Mosnodenvir in a DENV3 strain from genotype V. Finally, (vi) we screened all publicly available DENV1 and DENV4 sequences for the V91A mutation.

Research sample | Datasets used for the identification of V91A carrying sequences included 5434 sequences of DENV1, 4658 sequences of DENV2, 2189 datasets of DENV3, and 968 sequences of DENV4, they are meant to represent the known genetic diversity of DENV species. Datasets of non-recombinant sequences above 8500 nucleotides in length used for phylogenetic inference of past V91A emergence events comprised 4370 sequences for DENV2 and 1300 sequences for DENV3, they are meant to represent the known genetic diversity of DENV species suitable for phylogenetic inference. We also generated a set comprising exclusively DENV2-II sequences (length > 8500 nt, 1828) to identify the closest phylogenetic relatives of the French Caribbean Islands lineage (meant to represent the entire known diversity within DENV2-II genotype) , a set of 69 sequences to estimate the date of emergence of the V91A lineage linked to the epidemic (meant to represent the diversity within the V91A-carrying lineage associated with the French Caribbean epidemic suitable for inferring a time-resolved phylogeny), and a set corresponding to the 99 sequences from the French Caribbean Islands current epidemic available on Genbank as of March 2024 (meant to represent DENV circulating diversity during the French Caribbean Islands epidemic). To ensure the comprehensiveness of our DENV-2 dataset, we also included a set of 781 DENV-2 sequences from GISAID in our analysis.
For experimental testing, we included clinical isolates from the main French Caribbean Islands except Saint Martin because none was available at the time of the experiment, and included strains from both the year 2023 and 2024 in order to best represent the diversity of strains that circulated during the epidemic.

Sampling strategy | We filtered the data by: (i) excluding sequences that did not belong to DENV species, (ii) excluding sequences corresponding to patents, recombinants, clones.

Data collection | We collected all DENV sequences available on Genbank before November 2023 (DENV1 and DENV4), December 2023 (DENV3),

Data collection	March 2024 (DENV2). Also, we collected all DENV-2 data available on GISAID up to March 2024. Accession numbers of genomic DENV sequences used in all analyses are listed in the alignment files available at https://github.com/Snseli/Dengue_resistance_supplementary except for GISAID entries for which accessions are available in supplementary table 6.
Timing and spatial scale	We used genomic sequences for DENV produced globally from 1944 up to March 2024.
Data exclusions	Sequences that were too partial or for which genetic divergence is greater than what would have been expected based on the time of sampling.
Reproducibility	Two or more independent experiments (in triplicates) were performed for all in vitro experiments.
Randomization	There was no randomization
Blinding	Not applicable.

Did the study involve field work? ☐ Yes ☒ No

Reporting for specific materials, systems and methods

We require information from authors about some types of materials, experimental systems and methods used in many studies. Here, indicate whether each material, system or method listed is relevant to your study. If you are not sure if a list item applies to your research, read the appropriate section before selecting a response.

Materials & experimental systems

n/a	Involved in the study
☒	☐ Antibodies
☐	☒ Eukaryotic cell lines
☒	☐ Palaeontology and archaeology
☒	☐ Animals and other organisms
☒	☐ Clinical data
☒	☐ Dual use research of concern
☒	☐ Plants

Methods

n/a	Involved in the study
☒	☐ ChIP-seq
☒	☐ Flow cytometry
☒	☐ MRI-based neuroimaging

Eukaryotic cell lines

Policy information about cell lines and Sex and Gender in Research

Cell line source(s)	Monkey African Green kidney cells (Vero; ECACC CL 84113001; Vero E6: ATCC CRL-1586), C6/36 mosquito cells (from Aedes albopictus; ATCC CCL-1660).
Authentication	Cell lines were not authenticated.
Mycoplasma contamination	All cell lines tested negative for mycoplasma contamination.
Commonly misidentified lines (See ICLAC register)	None of the commonly misidentified cell lines were used.

Plants

Seed stocks	*Report on the source of all seed stocks or other plant material used. If applicable, state the seed stock centre and catalogue number. If plant specimens were collected from the field, describe the collection location, date and sampling procedures.*
Novel plant genotypes	*Describe the methods by which all novel plant genotypes were produced. This includes those generated by transgenic approaches, gene editing, chemical/radiation-based mutagenesis and hybridization. For transgenic lines, describe the transformation method, the number of independent lines analyzed and the generation upon which experiments were performed. For gene-edited lines, describe the editor used, the endogenous sequence targeted for editing, the targeting guide RNA sequence (if applicable) and how the editor was applied.*
Authentication	*Describe any authentication procedures for each seed stock used or novel genotype generated. Describe any experiments used to assess the effect of a mutation and, where applicable, how potential secondary effects (e.g. second site T-DNA insertions, mosiacism, off-target gene editing) were examined.*
